# Use of contemporary biomaterials in chronic osteomyelitis treatment: Clinical lessons learned and literature review

**DOI:** 10.1002/jor.24896

**Published:** 2020-11-04

**Authors:** Jan A. P. Geurts, Tom A. G. van Vugt, Jacobus J. C. Arts

**Affiliations:** ^1^ Maastricht University Medical Center Maastricht The Netherlands; ^2^ Department of Orthopedic Surgery Maastricht University Medical Center Maastricht The Netherlands

**Keywords:** biomaterials, infection

## Abstract

Chronic osteomyelitis has always been a therapeutic challenge for patient and surgeon due to the specific problems related with bone infection and bacterial biofilm eradication. Other than being the cause of infection or facilitating spread or persistence of infection, biomaterials are also becoming a tool in the treatment of infection. Certain novel biomaterials have unique and ideal properties that render them perfectly suited to combat infection and are therefore used more and more in the treatment of chronic bone infections. In case of infection treatment, there is still debate whether these properties should be focused on bone regeneration and/or their antimicrobial properties. These properties will be of even greater importance with the challenge of emerging antimicrobial resistance. This review highlights indications for use and specific material properties of some commonly used contemporary biomaterials for this indication as well as clinical experience and a literature overview.

## 1 INTRODUCTION

In orthopedic surgery and traumatology, bone infection is an underestimated and challenging condition for both the patient and the physician. Diagnosis can be difficult,^1^ treatment is often prolonged and cumbersome, sometimes involving multiple surgeries and can impose a significant financial burden on both the patient and the health system in general. Although tremendous progress has been made in the treatment of musculoskeletal infection over the years, studies have shown that elective surgery infection rates are not able to be reduced below 1–2% and failures of revision surgery remain as high as 33%.^2^, ^3^ The cost of treating bone infection is substantial and will increase as the absolute number of patients suffering from it keeps rising.^4^

Two specific entities of orthopedic infection can be identified: those infections that only involve bone (osteitis/osteomyelitis) and those affecting bone and an associated implant, like a joint replacement or some kind of osteosynthesis. Both entities are different in their approach, although overlap exists. To improve treatment outcomes, biomaterials have been used to help eradicate infection, fill bony defects and support remaining bone and/or implants. Some biomaterials function as antibiotic‐delivery devices, such as gentamicin‐loaded beads or spacers, as developed by Wahlig and Dingeldein in the 70s.^5^ Locally, they release high doses of antibiotics, far higher than the minimal inhibitory concentration (MIC) and higher than what can be achieved by parenteral administration of the same antibiotic, thereby eradicating an important part of the local bacterial load. These antibiotic‐loaded bone cements have served well over time, although several concerns have been addressed like antibiotic elution levels becoming subtherapeutic, thereby possibly inducing antimicrobial resistance, the absence of standardized formulation protocols and the absence of validated assays to determine the minimum biofilm eradication concentration to predict efficacy of these antibiotic‐loaded bone cements against specific microorganisms.^6^

Other materials have also been shown to have antibacterial properties and are used to coat the surface of an implant like nanoparticles, such as silver (Ag), magnesium (Mg), copper (Cu), and gold (Au) to prevent infection (by inhibiting the surface to be colonized by bacteria, who would than outrun host‐cells in the race for the surface).^7^, ^8^, ^9^ This is the concept, first described by Gristina in 1987, whereby when any foreign material is introduced in the body, a “race” will occur between our own cells/immune system and the microorganisms.^10^, ^11^ If the implant is covered by human or eukaryotic cells first, it will be “shielded” and as such be more difficult to reach for microorganisms. Eventually, (osseo)integration of the implant in the surrounding tissues will occur. On the other hand, if microorganisms are first, the implant will be contaminated. As soon as bacteria or other microorganisms bond with the surface, they will form biofilm, rendering themselves much more resistant to the body's immune system. This is because our immune cells cannot easily penetrate this biofilm and because bacteria downregulate their metabolism so they do not duplicate as often (metabolically less active), compared to their planktonic (or free‐floating) counterparts. The latter is also the reason why antibiotics are less effective for bacteria in biofilm, with MICs that can be 1000‐fold higher.^12^, ^13^ So, in essence bacteriae cover themselves in a slime layer when adhering on an implant, but when looking closer, biofilm is much more complex and concepts like metabolism, growth rate, gene expression changes, or persistor cells have to be taken into account.

Coating technology and implant modification (for instance: biomaterials with empirical antimicrobial behavior) to combat biofilm formation and/or persistence still deal with several concerns and necessitate further research, but will become important future methods to deal with implant‐related infection.^14^ Because of this, a separate working group was established at the 2018 International Consensus Meeting on Musculoskeletal Infection to provide insights on the biomaterial surface question.^15^

This review will focus on the use of contemporary biomaterials when dealing specifically with nonimplant related bone infection or chronic osteomyelitis. Important to note is that there is high variance in clinical level of evidence and level of efficacy between biomaterials used in osteomyelitis treatment. There is paucity of studies with high methodological quality.

Osteomyelitis is an infection of the bone or bone marrow without associated implant material and is most often caused by bacteria (*Staphylococcus aureus* being the most common) or fungi. It can be associated with open fracture surgery, bone reconstruction surgery or it can be caused by hematogenous spread of bacteria from another focus. If not treated properly, osteomyelitis can become chronic and be associated with delayed union or nonunion of fractures or failure of prosthesis implantation. In more severe untreated osteomyelitis bone sequestration, sinus formation or sepsis can cause disabling or life threatening complications.^16^ Acute osteomyelitis is most often seen in children (with open growth plates) and also not in the scope of this review.

**Figure 1:**
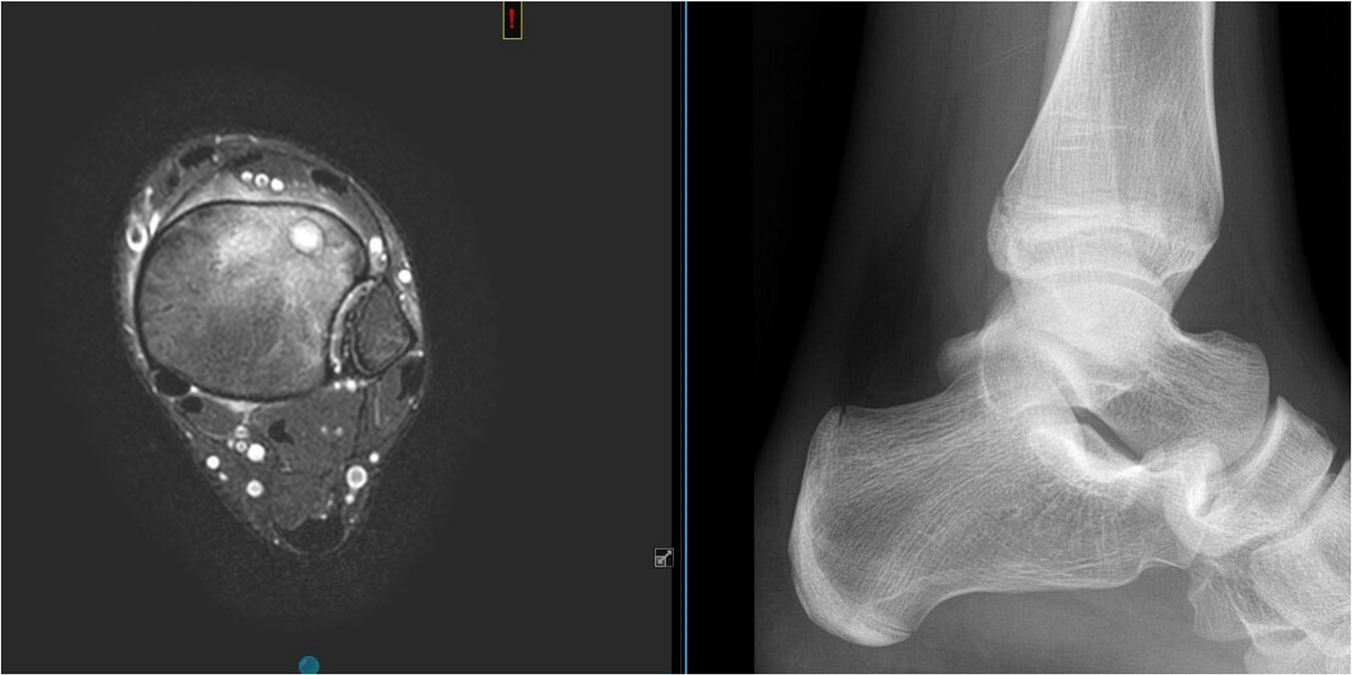
MRI and radiographic view of a Cierny I endomedullary osteomyelitis of the distal tibia (anterolateral). MRI, magnetic resonance imaging [Color figure can be viewed at wileyonlinelibrary.com]

**Figure 2:**
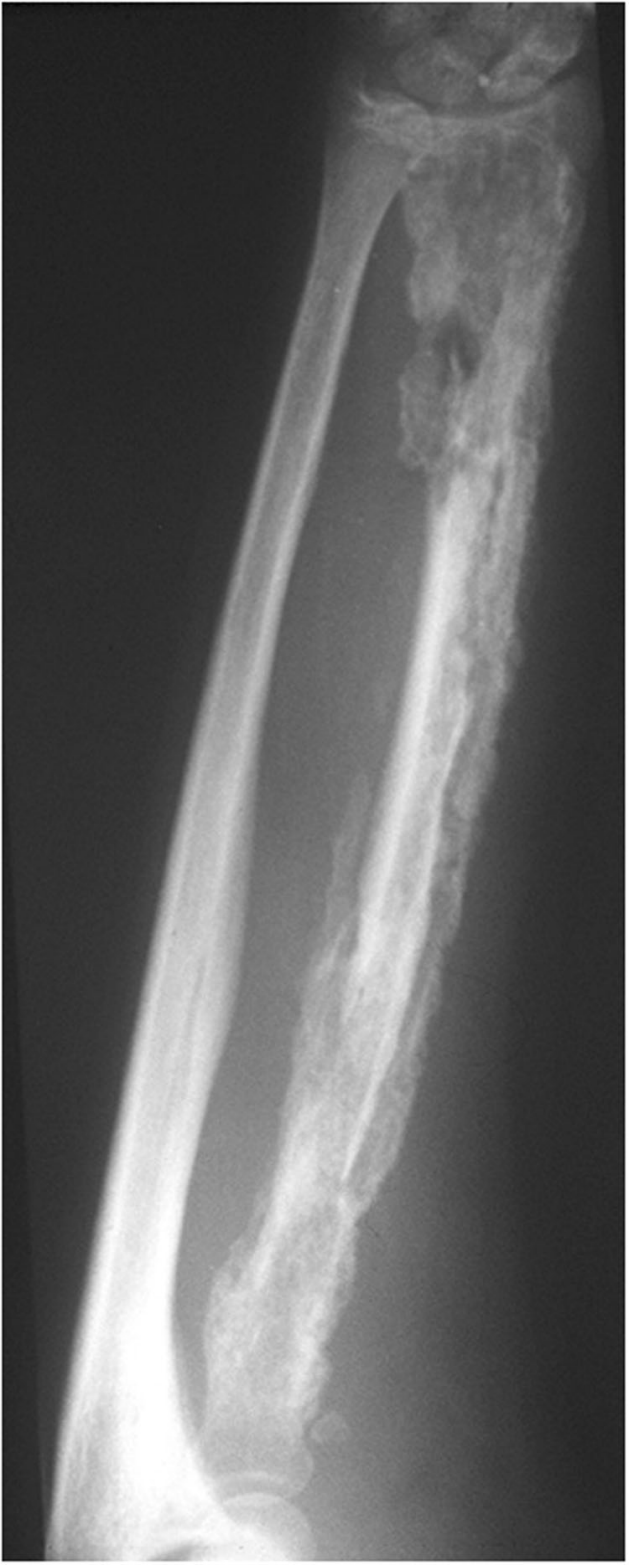
Chronic osteomyelitis of the radial bone, showing complete involvement of cortex and intramedullary canal (Cierny IV)

To optimally treat chronic osteomyelitis, a classification was developed by Cierny and Mader in 1985, which is still being extensively used to date.^17^ Stage I osteomyelitis is in essence an isolated infection of the endomedullary bone (“endosteal”) (Figure 1). Stage II osteomyelitis comprises the surface of the bone (“superficial”), which is affected by contiguous infection of surrounding soft tissues. Stage III is “localized,” meaning full‐thickness involvement of the bony cortex, but with the bone itself remaining stable. Finally, stage IV is “diffuse” osteomyelitis, meaning permeative, circumferential disease of hard (bone), and soft tissue. Before or after debridement, the bone will be unstable. Examples of the latter are infected nonunions, end‐stage septic joints, and through‐and‐through metaphyseal/epiphyseal lesions of the long bones (Figure 2).

Treatment of all types of chronic osteomyelitis consists of a combination of surgery and antibiotics (local and/or systemic). Surgery (debridement) diminishes most of the bacterial load in the affected area and will optimize surrounding soft tissues and vascularization, and is always the most important factor to achieve favorable outcome. Systemic and local antibiotics are aimed at eradicating remaining bacteria, adjusted to the results of the culture specimens taken. Biomaterial choice per case should be based on assessment of local mechanical and biological environment demands, mechanical properties of the biomaterial itself, and antimicrobial eradication demands.

In the last decades antibiotic loaded polymethyl methacrylate (PMMA) beads have been used in clinical treatment of chronic osteomyelitis in a two‐stage fashion. Due to the availability of biodegradable antibiotic loaded bone graft substitutes, a combination of local treatment and one‐stage principle was introduced. There are multiple commercially available biodegradable biomaterials that are studied for treatment of chronic osteomyelitis in a one‐stage fashion (Table 1). These materials are generally based on antibiotic loaded calcium sulfates, calcium phosphates, or bioactive glasses.

**Table 1:** Properties of commercially available and clinical used biomaterials suitable for treatment of chronic osteomyelitis

Product name	Composition	Antimicrobial mechanism	Antibiotic type	Level of evidence^a^
BonAlive^®^	S53P4 bioactive glass S53P4 (53% SiO_2_, 4% P_2_O_5_, 23% Na_2_O, and 20% CaO)	Release of surface ions causing increase of pH and osmotic pressure	None	2b
Cerament G/V^®^	60% calcium sulfate, 40% hydroxyapatite	Antibiotic loaded BGS	Gentamicin, vancomycin	2b
Herafill‐G^®^	Calcium sulfate and calcium carbonate	Antibiotic loaded BGS	Gentamicin	3b
Osteoset‐T^®^	ɑ‐Hemihydrate calcium sulfate	Antibiotic loaded BGS	Tobramycin	2b
Perossal^®^	Nano‐crystalline hydroxyapatite (51.5%) and calcium sulfate (48.5%)	Antibiotic loaded BGS	Different types of antibiotics (surgeon's choice)	2b
Stimulan^®^	Hemihydrate calcium sulfate	Antibiotic loaded BGS	Gentamicin, vancomycin, tobramycin	2b

Abbreviations: BGS, bone graft substitute; CEBM, Centre of Evidence Based Medicine.

a Level of evidence is based on best available methodological quality and is based on the CEBM criteria of the University of Oxford centre of evidence (2b, individual cohort study (including low quality RCT; e.g., <80% follow‐up; 3b, individual case‐control study).

Important to note is that biomaterials/antibiotics are often used off‐label in infection treatment and that high quality studies/randomized controlled trial (RCT) data are often not available. As such their mechanism of action or efficacy can often be questioned.

The antibiotic loaded calcium based biomaterials have a completely different mechanism of antibacterial and osteoconductive/osteostimulative activity in comparison to the S53P4 bioactive glass. Antibacterial activity is based on the generally known pathways of used antibiotics (commonly gentamicin, vancomycin, or tobramycin). New bone formation is based on osteoconductivity and osteoinductivity after relatively fast degradation of these materials resulting the formation of a bone mineral scaffold.

The bone‐bonding capacity and antibacterial properties of S53P4 bioactive glass are completely different to the abovementioned biocomposites. In addition, these are properties not seen in any other commercially available bioactive glass to date.^18^, ^19^, ^20^, ^21^ The mechanism responsible for this feature is twofold:

1. When in contact with bodily fluids, the glass granules are wetted and sodium (Na) is released from the glass surface. As a result, pH rises locally (environment becoming more alkaline). This is an unfavorable situation for bacteria in which their cell wall disintegrates and the bacterium”bursts.”
2. Other ions released from the surface (Na, Ca, P, Si) cause a local rise in osmotic pressure, again rendering the environment unsuitable for bacterial growth (initially dehydration, downregulation of DNA replication and upregulation of starvation genes, inhibition of proliferation, and cell wall failure).^22^

These discussed biomaterials can be used in treatment of the different types of osteomyelitis and these will now be addressed, according to the Cierny‐classified type of chronic osteomyelitis.

## 2 CIERNY STAGE I OSTEOMYELITIS

Treatment of Stage I osteomyelitis of a long bone consists of debridement of the endomedullary canal, either by reaming from proximal or distal or curettage through a cortical window. Obviously, meticulous culture specimens have to be taken to know the causative germ (and this is evidently applicable for Cierny II–IV cases too) and adapt adjuvant systemic antibiotic therapy or antibiotics administered in graft or other biomaterials. For this, the authors adhere to the “Oxford protocol” which consists of separate instruments for each sample, no‐touch technique, a minimum of three samples and no suction until samples are taken.^21^, ^23^, ^24^ After mechanical debridement, a thorough wash‐out of the canal is performed and importantly in all osteomyelitis treatment: management of dead space, meaning the endomedullary canal has to be filled/obliterated to prevent formation of residual hematoma. Currently used and recommended biomaterials suitable for this purpose are resorbable calcium sulfate and/or calcium phosphate pellets (these are brittle and dissolve and can therefore not serve any structural supporting function, only void filling) and antibiotic‐impregnated PMMA beads which elute high levels of antibiotics locally for 2–4 weeks. Calcium sulfate/phosphate pellets are biodegradable, which enable a “one‐stage” operative procedure, versus the PMMA beads that are nonresorbable, requiring a “two‐stage” procedure, to remove the beads in a second procedure.^23^, ^25^ The bony bed can then be grafted by cancellous or vascularized bone‐graft to obliterate the dead space. One‐stage revision surgery is preferred over a two‐stage procedure, because of the lower burden to the patients, shorter hospitalization, lower infection risks, improved joint function, and lower costs.^26^ Drawbacks of one‐stage surgery are the sometimes lower success rates and thereby it is not always possible to accurately plan the bone reconstruction part of the surgery.^27^, ^28^

Examples of calcium sulfate (Ca‐S) or calcium phosphate (Ca‐P) or hybrid formulation pellets available for this indication are Herafill‐G^®^ (Heraeus Medical GmbH), Osteoset‐T^®^ (Wright Medical Technology), PerOssal^®^ (Osartis GmbH), and Stimulan^®^ (Biocomposites).

Fleiter et al.^29^ reported an 80% infection eradication rate in a series of 20 osteomyelitis patients treated with Herafill‐G^®^. In another paper, Franseschini describes a successful outcome of treatment in a patient with the use of Herafill‐G^®^ beads in combination with nanocrystalline carbonated hydroxyapatite of bovine origin and a hemisoleus flap.^30^

Results of the usage of PerOssal^®^ were reported by Berner in 2008 and Von Stechow in 2009.^31^, ^32^ The latter (case series; no control group) reporting its use in 19 patients with spondylodiscitis, with successful outcome at 1 year in 12. Visani described a healing rate of 86.5% in 52 patients with a minimum follow up of 24 months and if recurrence occurred, it happened 106 days later than in the group not treated with PerOssal^®^ (45 patients).^33^

Experience with Osteoset‐T^®^ (which is tobramycin‐impregnated) with good level of evidence was reported by McKee in 2010: infection eradication in 86% (12 out of 14 patients) with a mean follow‐up of38 months (RCT).^34^ In a retrospective review, 21 cases of chronic tibial osteomyelitis treated with Osteoset‐T^®^ were reported by Humm et al.^35^ with infection eradication in 20 of 21 patients, mean time of follow‐up being 16 months.

Finally, Gauland^36^ reported on the use of Stimulan^®^ in managing lower extremity osteomyelitis showing encouraging results without the use of oral and/or intravenous antibiotics. The results showed 279 of 323 patients (86.4%) clinically healed with no recurrence of osteomyelitis to any specific anatomical location in the longest follow‐up period of 5.5 years.

Important to note is that all mentioned studies did not exclusively address Cierny I osteomyelitis patients, but the tendency is to treat Cierny I osteomyelitis with these kind of easy‐to‐use, biodegradable, antibiotic‐eluding biomaterials.

## 3 CIERNY STAGE II OSTEOMYELITIS

This type of chronic osteomyelitis is in essence a bone infection, contiguous with an overlying soft tissue infection, in other words: bone at the base of a soft‐tissue wound. Treatment consists of addressment of the soft tissue problem by debridement, resection of all necrotic tissues and abundant lavage. The bone can be debrided by resection of the exposed cortex, thereby taking care not to breach the full cortical thickness (which would cause intramedullary bacterial contamination and potential biomechanical instability of fracture risk). Debridement is complete when healthy bleeding cortical bone is at the base of the wound. Preferably this is done with chisels, osteotomes or nibblers. High‐speed burs should be avoided, as they often cause thermal necrosis of the bone, which is not beneficial for the final outcome as dead bone again is the perfect target for bacterial colonization. Additional biomaterials are seldom indicated in this type of osteomyelitis as dead space is addressed by local soft tissue coverage.

## 4 CIERNY STAGE III AND IV OSTEOMYELITIS

Cierny III and IV are addressed together, because debridement will most often result in extensive bone and soft tissue loss, which requires some form of stabilization of the involved bone. In some cases, the bone can be acutely shortened to address the osseous defect, but otherwise a more or less larger defect has to be filled and stabilized. Small defects can be filled with a local myoplasty, meaning local muscle is used to fill the defect, but more often defects are larger or more complex so alternatives have to be used. One‐ or two‐stage procedures are common, with or without use of implants or external fixation for added stability. Two‐stage procedures used to be the golden standard and involved debridement in a first stage, with void filling by means of nonresorbable PMMA beads. Patients would then receive a course of antibiotics for 6–8 weeks and once infection resolved, the PMMA beads would be removed in a second operation and the bony defect filled with autograft, allograft, or some other synthetic biomaterial. Two‐stage technique, however has not been abandoned completely, as it is part of the Masquelet (or “induced membrane”) technique which is used often when dealing with larger (>6 cm) segmental bone defects, because (auto‐ or allo)grafting alone will not be able to heal these defects, as resorption of the graft will occur, even within a well‐vascularized muscle envelope.^37^ In a first stage, debridement of infected bone will be thoroughly performed, ending the procedure with a gap between healthy bony ends that will then be filled with a PMMA cement spacer (with or without antibiotics), and soft tissues are addressed properly. This cement spacer will provoke a “foreign body reaction” by the adjacent soft tissue, which will try to encapsulate it by forming a highly vascularized pseudo membrane (also called induced membrane). After 6–8 weeks, the spacer is removed, with carefully sparing the induced membrane and filling the “chamber” with morcelized autograft (as initially described) or a contemporary biomaterial. Essential throughout the whole treatment is adequate stable fixation of the affected long bone. The effectiveness of this technique has been shown in several studies.^38^

Two commonly used contemporary biomaterials in these more complex (one‐stage and/or Masquelet technique) cases are the previously discussed bioactive glass S53P4 (Bonalive^®^; Bonalive Biomaterials Ltd.) and a gentamicin‐loaded calcium sulfate/hydroxyapatite biocomposite (Cerament G^®^; Bonesupport).

Concerning S53P4, both previously mentioned mechanisms prevent adhesion of bacteria onto the granules as well as bacterial growth in the vicinity (bactericidal effect). To date, no bacterial resistance to S53P4 bioactive glass has been reported and Drago et al. showed that S53P4 bioactive glass is even active against multidrug resistant bacterial strains.^39^, ^40^ There is also some very preliminary data on activity of S53P4 bioactive glass against Candida albicans, a fungus responsible for some bone infections.^41^, ^42^ Interestingly, both mechanisms are detrimental for prokaryotic structures, like bacteria, but not so for eukaryotic cells.^43^, ^44^

The bone bonding properties are initiated by the same release of ions, transforming the glass surface chemically into a state resembling the mineral phase of normal bone (silica‐gel layer). This silica‐gel layer acts as a template for calcium phosphate (CaP) precipitation, which in turn crystallizes to a hydroxyapatite surface (the main component of natural bone), activating specific osteoblast genes, thereby stimulating their recruitment and differentiation.^44^ The glass particles are also osteoconductive, serving as a scaffold for bone on growth. Eventually, over the course of several years, the particles will dissolve completely, being replaced by natural bone. The whole of this process has been named “osteostimulation,” signifying that bioactive glass granules stimulate the recruitment and differentiation of osteoblasts, activate osteoblasts to produce new bone and activate specific osteoblast genes as a response to ion dissolution from the material. Osteostimulative materials need a bony environment to stimulate new bone formation. Therefore, the most ideal defect to fill with bioactive glass is a contained defect. Finally, there is growing evidence that bioactive glass S53P4 also has angiogenetic properties, meaning it stimulates formation of new vascular structures (blood vessels) that in turn is a prerequisite for healthy bone formation.^45^

Good results with the use of S53P4 have been reported in a multicenter study by Lindfors et al.^46^ in 116 patients with a cure rate of 90% (median follow‐up 31 months).

A good alternative for one‐stage treatment of osteomyelitic defects is the gentamicin‐loaded calcium sulfate/hydroxyapatite biocomposite Cerament G^®^. This biomaterial contains 17.5 mg/ml of gentamicin as a paste which been designed to have a neutral pH (7.0–7.2). Mixing and injection devices ensure a homogenous distribution of antibiotic, while the material properties of Cerament G mean that this entire antibiotic is made available locally in high dosage. This was confirmed by several in vitro elution studies, not only for gentamicin, but also for other antibiotics like vancomycin, tobramycin, rifampicine, piperacillin‐tazobactam, ceftazidime, all of which can be beneficial when treating for instance methicillin‐resistant *S. aureus* and *Pseudomonas auruginosa*.^47^ The biocomposite itself promotes bone healing by stimulating de novo bone formation and remodeling into host bone in 6–12 months. It is the combination of calcium sulfate with hydroxyapatite, which makes this biomaterial osteoconductive, while also preserving sufficient strength.^48^ Tissue response towards the material is also enhanced when compared to hydroxyapatite alone.

Good outcomes have been reported by McNally et al.^49^ in a prospective series of 100 patients, including 71 fracture related infections, with eradication of infection in 96%.

What has to be taken into consideration when using any of these described biomaterials are defect location, defect size, contained or uncontained bony defects, weight bearing area of the bone or not, and so forth. Therefore, any of these materials is not exclusively indicated for a certain osteomyelitis type, nor does it mean that several options exist for treating one specific osteomyelitis problem.

## 5 CONCLUSION

Several biomaterials are currently available to address bone defects caused by either bone infection itself or the treatment thereof by debridement until healthy tissue remains. The biomaterials can deliver local antibiotics and serve as scaffolds for native bone to regenerate. This has helped surgeons in dealing with chronic osteomyelitis, which has historically been a very difficult problem to tackle. In essence, a lot of these patients can now be treated in a one‐stage fashion, something that would not have been possible with these advanced biomaterials. However, it is clear that there is not one biomaterial that can solve all different types of chronic osteomyelitis. Future research and development will still be necessary to improve these materials, especially with a lot of these biomaterials relying on “classic” eradication of infection by application of antibiotics and the emerging antimicrobial resistance becoming more and more problematic.

## AUTHOR CONTRIBUTIONS

*Discussion paper content, draft of paper, acquisition and interpretation of data, final approval and submission*: Jan A. P. Geurts. *Discussion paper content, interpretation of data, critical revision*: Tom A. G. van Vugt. *Discussion paper content, interpretation of data, critical revision*: Jacobus J. C. Arts. All authors have read and approved the final submitted manuscript.
